# Antithrombotic Effects of the Novel Small-Molecule Factor XIa Inhibitor Milvexian in a Rabbit Arteriovenous Shunt Model of Venous Thrombosis

**DOI:** 10.1055/a-2061-3311

**Published:** 2023-04-24

**Authors:** Xinkang Wang, Qiu Li, Fuyong Du, Neetu Shukla, Andrea R. Nawrocki, Madhu Chintala

**Affiliations:** 1 Cardiovascular & Metabolism Therapeutic Area, Janssen Research & Development, LLC, Spring House, Pennsylvania, United States; 2 Formulation, Janssen Research & Development, LLC, Spring House, Pennsylvania, United States

**Keywords:** anticoagulants, factor XI, thrombosis, models, animal, pharmacology

## Abstract

**Background** Factor XIa (FXIa) is an emerging therapeutic target, and FXIa inhibition is a promising mechanism to improve therapeutic index over current anticoagulants. Milvexian (BMS-986177/JNJ-70033093) is an oral small-molecule FXIa inhibitor.

**Objective** Milvexian's antithrombotic efficacy was characterized in a rabbit arteriovenous (AV) shunt model of venous thrombosis and compared with the factor Xa inhibitor apixaban and the direct thrombin inhibitor dabigatran.

**Methods** The AV shunt model of thrombosis was conducted in anesthetized rabbits. Vehicle or drugs were administered as intravenous bolus plus a continuous infusion. Thrombus weight was the primary efficacy endpoint. Ex vivo activated partial thromboplastin time (aPTT), prothrombin time (PT), and thrombin time (TT) were measured as the pharmacodynamic responses.

**Results** Milvexian dose dependently reduced thrombus weights by 34.3 ± 7.9, 51.6 ± 6.8 ( *p* < 0.01; *n* = 5), and 66.9 ± 4.8% ( *p* < 0.001; *n* = 6) versus vehicle at 0.25 + 0.17, 1.0 + 0.67, and 4.0 ± 2.68 mg/kg bolus + mg/kg/h infusion, respectively. Ex vivo clotting data supported a dose-dependent prolongation of aPTT (with 1.54-, 2.23-, and 3.12-fold increases from baseline upon the AV shunt start), but no changes in PT and TT. Dose-dependent inhibition in thrombus weight and clotting assays was also demonstrated for both apixaban and dabigatran as the references for the model validation.

**Conclusion** Results demonstrate that milvexian is an effective anticoagulant for prevention of venous thrombosis in the rabbit model, which supports the utility of milvexian in venous thrombosis, as seen in the phase 2 clinical study.

## Graphical abstract

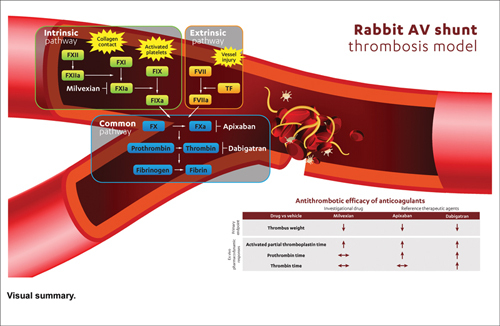

## Introduction

Thrombotic conditions, such as atrial fibrillation, stroke, myocardial infarction, deep vein thrombosis (DVT), and pulmonary embolism, are the leading causes of morbidity and mortality worldwide. 1 Current oral anticoagulants include vitamin K antagonists (warfarin) and direct oral anticoagulants that target serine proteases in the common coagulation pathway, such as thrombin (dabigatran) or factor Xa (FXa; apixaban, rivaroxaban, edoxaban, betrixaban). Although these anticoagulants are effective in treating and/or preventing thrombosis, all are associated with an increased risk of bleeding events, which results in patients either not receiving antithrombotic therapy or being suboptimally treated with currently available agents. 2 Thus, a significant unmet medical need remains for novel anticoagulants that can effectively prevent thrombosis while preserving hemostasis.

Factor XIa (FXIa), a serine protease within the intrinsic pathway of coagulation, has emerged as a new and promising drug target based on evidence that it inhibits the pathophysiologic formation of a thrombus while preserving normal hemostatic function. 3 4 5 Human hemophilia C patients who are severely deficient in factor XI (FXI) have a mild to moderate risk of bleeding and display reduced incidence of DVT and/or ischemic stroke. 6 7 On the other hand, elevated levels of FXI are associated with the risk of several thromboembolic disorders. 8 9 Furthermore, the synthetic antisense oligonucleotide IONIS-FXIRx that targets FXI mRNA proved to be superior to enoxaparin in reducing the incidence of DVT in a phase 2 total knee arthroplasty clinical trial and appeared to be safe with respect to bleeding. 10 Similarly, abelacimab, 11 a monoclonal antibody that binds to the catalytic domain of FXI and locks the zymogen, and osocimab, 12 a monoclonal antibody against FXIa, both demonstrated the antithrombotic efficacy in separate phase 2 clinical studies to prevent venous thromboembolism among patients undergoing knee arthroplasty.

Various preclinical studies demonstrated the role of FXI/FXIa in thrombosis, with minimal impact on hemostasis. In animal models, FXI-knockout mice are protected from arterial and venous thrombosis 13 14 15 without increasing bleeding time while also exhibiting prolonged activated partial thromboplastin time (aPTT) and normal prothrombin time (PT). 16 Similar observations were reported with specific inhibition of FXI/FXIa in rats, 17 rabbits, 18 19 and baboons. 20 21

Hence, FXIa inhibitors are promising novel anticoagulants that provide an improved therapeutic index over the currently approved oral anticoagulants. Milvexian is an oral, small-molecule FXIa inhibitor that has demonstrated robust efficacy in a phase 2 clinical study for the prevention of venous thromboembolism in patients undergoing total knee arthroplasty. 22 23 The efficacy of milvexian in a rabbit model of electrically mediated carotid arterial thrombosis (ECAT) was recently reported. 24 The current study reports on preclinical antithrombotic efficacy of milvexian in a rabbit arteriovenous (AV) shunt model of venous thrombosis, a model that was used to evaluate the in vivo efficacy of the FXa inhibitor apixaban 25 and the direct thrombin inhibitor dabigatran. 26 In the current study, the FXa inhibitor apixaban and the direct thrombin inhibitor dabigatran were used as reference agents.

## Materials and Methods

### Animals

Male New Zealand white rabbits were purchased from Charles River Lab (Wilmington, MA, United States). Rabbits with a body weight between 2.45 and 3.22 kg and aged about 10 to 14 weeks were used for the AV shunt study. All the animal care and experimental procedures were approved by the Institutional Animal Care and Use Committee (IACUC) of Janssen (Spring House, PA, United States). Animals were housed in standard rabbit cages, one animal per cage, and maintained at 22°C for a 12-hour daylight cycle. Standard rabbit diet and water were provided, along with enrichment with fruit and carrots, except for green leaves.

### Arteriovenous Shunt Protocol

The rabbit AV shunt model was established as described previously. 19 27 Rabbits were randomly divided into different experimental groups. Each group had five to six animals based on historic data power analysis.

Ketamine hydrochloride (HCl; 20.0–50.0 mg/kg intramuscular [IM]) and xylazine (2.0–10.0 mg/kg IM) were used for anesthetic induction. Subsequent anesthetic maintenance was provided as needed (i.e., approximately every 30–50 minutes) with ketamine HCl (22.7–25.0 mg/kg/∼0.5 h IM) and xylazine (2.3–5.0 mg/kg/∼0.5 h IM) for the duration of the study. Alternatively, rabbits were induced and subsequently maintained on isoflurane gas anesthetic (2–4% isoflurane and O _2_ flow rate at 0.9–1.2 L/min via nose cone). Anesthetic depth was continuously monitored throughout the study period via visual assessment (i.e., signs of wakefulness, body movement) and evaluation of pedal/palpebral reflexes, jaw tone, and/or changes in heart and/or respiratory rate. Anesthetic adjustments were made as needed to maintain a steady anesthetic plane. Vital signs (e.g., heart rate, electrocardiogram, oxygen saturation levels, arterial blood pressure, respiratory rate, body temperature) were monitored throughout the study period.

Following anesthesia, a 2-cm incision was made on each thigh to expose the femoral artery and vein, respectively. The vein and artery were cannulated with PE-100 tubing and allowed to equilibrate for 20 minutes. An AV shunt device was then connected between the femoral arterial (at one thigh) and femoral vein (at the opposite thigh) cannulas. The shunt device consisted of an outer piece of Tygon tubing (length: 8 cm; inner diameter [i.d.]: 7.9 mm) and an inner piece of tubing (length: 2.5 cm; i.d.: 4.8 mm). The shunt also contained an 8-cm length of 2–0 silk thread as the trigger for thrombus formation. Forty minutes after blood flow through the shunt was initiated, the shunt was disconnected and the silk thread, along with the clot, was removed and weighed.

Milvexian used in this study was synthesized by scientists at Bristol Myers Squibb (New Brunswick, NJ, United States). The compound structure, selectivity, and pharmacology profile of milvexian have been reported previously. 22 The doses of milvexian for the current study were selected based on a rabbit ECAT study 24 and increased one dose level for the rabbit AV shunt model. The loading dose and continuous infusion regimens aimed to achieve a steady-state plasma drug level during the course of AV shunt thrombosis. The milvexian dosing solutions for the current study were prepared fresh daily in a vehicle containing 70% polyethylene glycol and 10% ethanol with 20% H _2_ O at 0.42 mg/kg (0.25 mg/kg bolus + 0.17 mg/kg/h infusion), 1.67 mg/kg (1.0 mg/kg bolus + 0.67 mg/kg/h infusion), and 6.68 mg/kg (4.0 mg/kg bolus + 2.68 mg/kg/h infusion) in a dosing volume of 5 mL/3 kg body weight. Vehicle or various milvexian doses were administered intravenously (IV) via the marginal ear vein beginning 30 minutes prior to the AV shunt.

Apixaban was purchased from AstaTech (Catalog No: 41088; Bristol, PA, United States). The IV dosing regimen for apixaban in rabbits essentially followed a previously reported procedure, 25 28 29 with the following specifications: The apixaban dosing solution was prepared in vehicle (35% hydroxypropyl β-cyclodextrin in 10-mM phosphate buffer, pH 7.0) and dosed with bolus plus continuous IV infusion to achieve a steady-state plasma drug level during AV shunt thrombosis. Apixaban was dosed at 0.015 mg/kg (0.006 mg/kg bolus + 0.009 mg/kg/h infusion), 0.15 mg/kg (0.06 mg/kg bolus + 0.09 mg/kg/h infusion), and 1.5 mg/kg (0.6 mg/kg bolus + 0.9 mg/kg/h infusion) IV in a volume of 2 mL/kg.

Dabigatran was purchased from PharmaBlock Sciences Nanjing Inc (Catalog No: PBN20120440; Nanjing, China). The dosing solution was prepared in vehicle containing 10% N-N-dimethylacetamide:90% of 5% dextrose. The doses for the current study were selected based on a previous report 29 at 0.015 mg/kg (0.006 mg/kg bolus + 0.009 mg/kg/h infusion), 0.15 mg/kg (0.06 mg/kg bolus + 0.09 mg/kg/h infusion), and 1.5 mg/kg (0.6 mg/kg bolus + 0.9 mg/kg/h infusion) IV in a volume of 2 mL/kg.

### Ex Vivo Clotting Assays

Coagulation reagents were purchased from Diagnostica Stago (Parsippany, NJ, United States), namely, thrombin 10 (Catalog No: 00611; Lot No: 251851) for thrombin time (TT), Neoplastine Cl 5 (Catalog No: 00666; Lot No: 01504) for PT, and C.K. Prest 5 (Catalog No: 00597; Lot No: 112768) and CaCl _2_ (0.025 M; Catalog No: 00367; Lot No: 254158) for aPTT. Coagulometer STA rt4 (Diagnostica Stago) was used for ex vivo clotting time analysis.

Blood samples (1 mL each) were collected from the femoral artery cannula in 3.2% sodium citrate (BD Biosciences, Franklin Lakes, NJ, United States) before and after shunt setup and centrifuged at 2,500 g for 15 minutes at 20°C. The resulting platelet-poor plasma (PPP) was transferred into clean tubes and stored at –80°C until use.

Coagulation assays were performed with PPP in a four-channel coagulometer STA rt4 (Diagnostica Stago). Briefly, for the aPTT assay, 50-µL C.K. Prest reagent was incubated with 50-µL PPP for 3 minutes at 37°C in a cuvette, and coagulation was initiated by adding 50-µL 25-mM CaCl _2_ . For the PT assay, 50-µL PPP was incubated for 1 minute at 37°C in a cuvette, and coagulation was initiated by adding 100-µL Neoplastine Cl reagent. For the TT assay, 100-µL PPP was incubated for 1 minute at 37°C in a cuvette, and coagulation was initiated by adding 100-µL thrombin (1.5 IU/mL). The onset of clotting was recorded as the coagulation time. The above clotting assays were performed on blood samples from each animal.

### Statistical Analysis

All data are expressed as mean ± standard error of the mean. Statistical analyses were performed using Prism software (v8.0; GraphPad, San Diego, CA, United States). Significance was defined at *p* <0.05, as determined by one-way analysis of variance. Tukey's test was used for multiple comparisons.

## Results

**Fig. 1:**
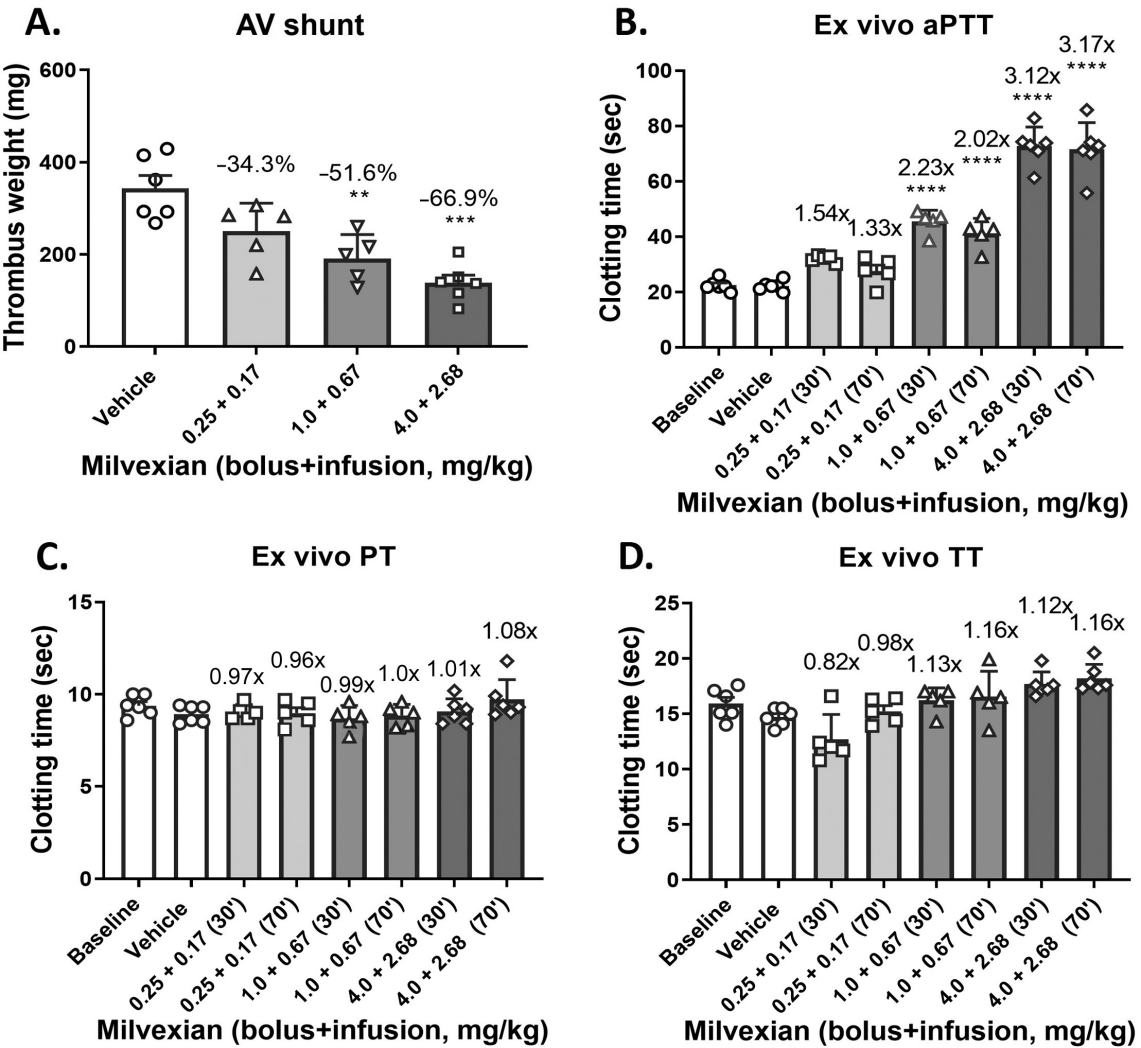
Dose-dependent effects of milvexian on thrombus formation in the rabbit arteriovenous (AV) shunt model of thrombosis and ex vivo clotting. ( **A** ) Milvexian dose dependently reduced thrombus weight in the rabbit AV shunt model of thrombosis, with 34.3 ± 7.9, 51.6 ± 6.8, and 66.9 ± 4.8% inhibition versus vehicle, respectively, at doses of 0.25 + 0.17, 1.0 + 0.67, and 4.0 + 2.68 mg/kg. Plasma samples were taken immediately prior to (30 minutes after infusion) and after (at 70 minutes) the AV shunt thrombosis and subjected to the ex vivo analysis of ( **B** ) activated partial thromboplastin time (aPTT), ( **C** ) prothrombin time (PT), and ( **D** ) thrombin time (TT). Each symbol in ( **A** ) represents 1 animal. Fold changes are indicated against vehicle in ( **B** ) and ( **C** ). ** *p* < 0.01. *** *p* < 0.001. **** *p* < 0.0001. Analysis of variance (ANOVA) followed by Tukey's test.

The dose-dependent effects of milvexian on thrombus weight in the rabbit AV shunt thrombosis model were evaluated, and the data are illustrated in Fig. 1 . Milvexian significantly reduced thrombus weight in the rabbit AV shunt model of thrombosis by 34.3 ± 7.9, 51.6 ± 6.8 ( *p* <0.01; *n* = 5), and 66.9 ± 4.8% ( *p* <0.001; *n* = 6) at 0.25 + 0.17, 1.0 + 0.67, and 4.0 + 2.68 mg/kg milvexian of bolus plus infusion doses, respectively ( Fig. 1A ).

Blood samples collected at 30 and 70 minutes after bolus injection plus initiation of infusion of milvexian (i.e., immediately prior to and after AV shunt thrombosis) were used for clotting assays. Ex vivo clotting data are summarized in Fig. 1 . Milvexian produced a dose-dependent prolongation of aPTT ( Fig. 1B ), with 1.54-, 2.23- ( *p* <0.001), and 3.12-fold ( *p* <0.001) increases from baseline upon the AV shunt start, respectively. No significant changes were observed for milvexian in PT ( Fig. 1C ) and TT ( Fig. 1D ) in the ex vivo clotting assays.

**Fig. 2:**
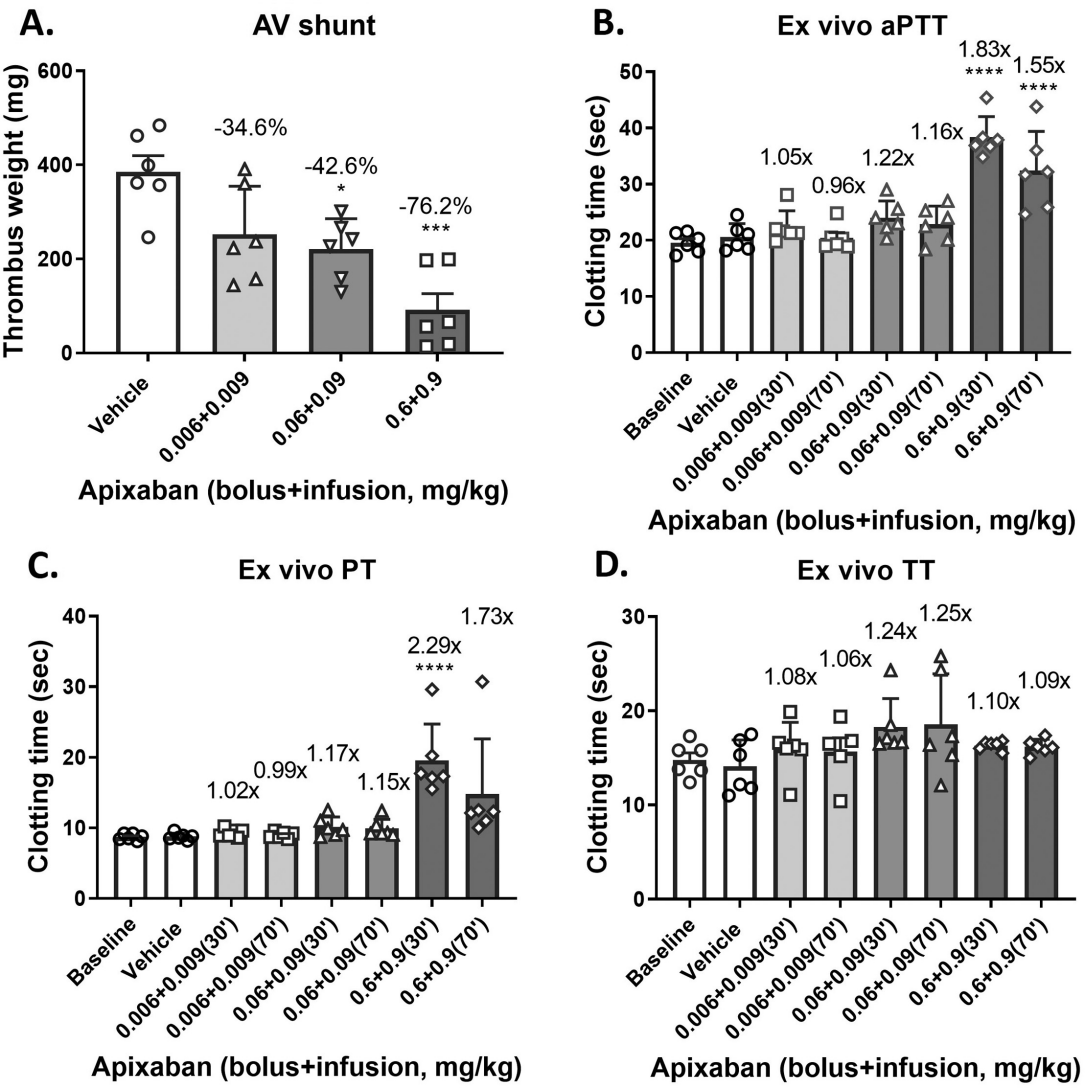
Dose-dependent effects of apixaban on thrombus formation in the rabbit arteriovenous (AV) shunt model of thrombosis and ex vivo clotting. ( **A** ) Apixaban dose dependently reduced thrombus weight in the rabbit AV shunt model of thrombosis. Plasma samples were taken immediately prior to (30 minutes after infusion) and after (at 70 minutes) the AV shunt thrombosis and subjected to the ex vivo analysis of ( **B** ) activated partial thromboplastin time (aPTT), ( **C** ) prothrombin time (PT), and ( **D** ) thrombin time (TT). Each symbol in ( **A** ) represents 1 animal. Fold changes are indicated against vehicle in panels ( **B** ) and ( **C** ). * *p* <0.05. *** *p* < 0.001. **** *p* < 0.0001. Analysis of variance (ANOVA) followed by Tukey's test.

Both apixaban and dabigatran were used to calibrate the rabbit AV shunt model. Apixaban dose dependently reduced thrombus weight in the rabbit AV shunt model of thrombosis, with 34.6 ± 10.9, 42.6 ± 6.9 ( *p* < 0.05; *n* = 6), and 76.2 ± 9.0% ( *p* < 0.001; *n* = 6) inhibition versus vehicle, respectively, at doses of 0.006 + 0.009, 0.06 + 0.09, and 0.6 + 0.9 mg/kg of bolus plus infusion as summarized in Fig. 2A . Ex vivo clotting assays showed significant increases for apixaban in aPTT (1.83-fold; *p* < 0.0001) and PT (2.29-fold; *p* < 0.0001) at the 0.6 + 0.9 mg/kg dose ( Fig. 2B and 2C ), but no significant change for TT ( Fig. 2D ).

**Fig. 3:**
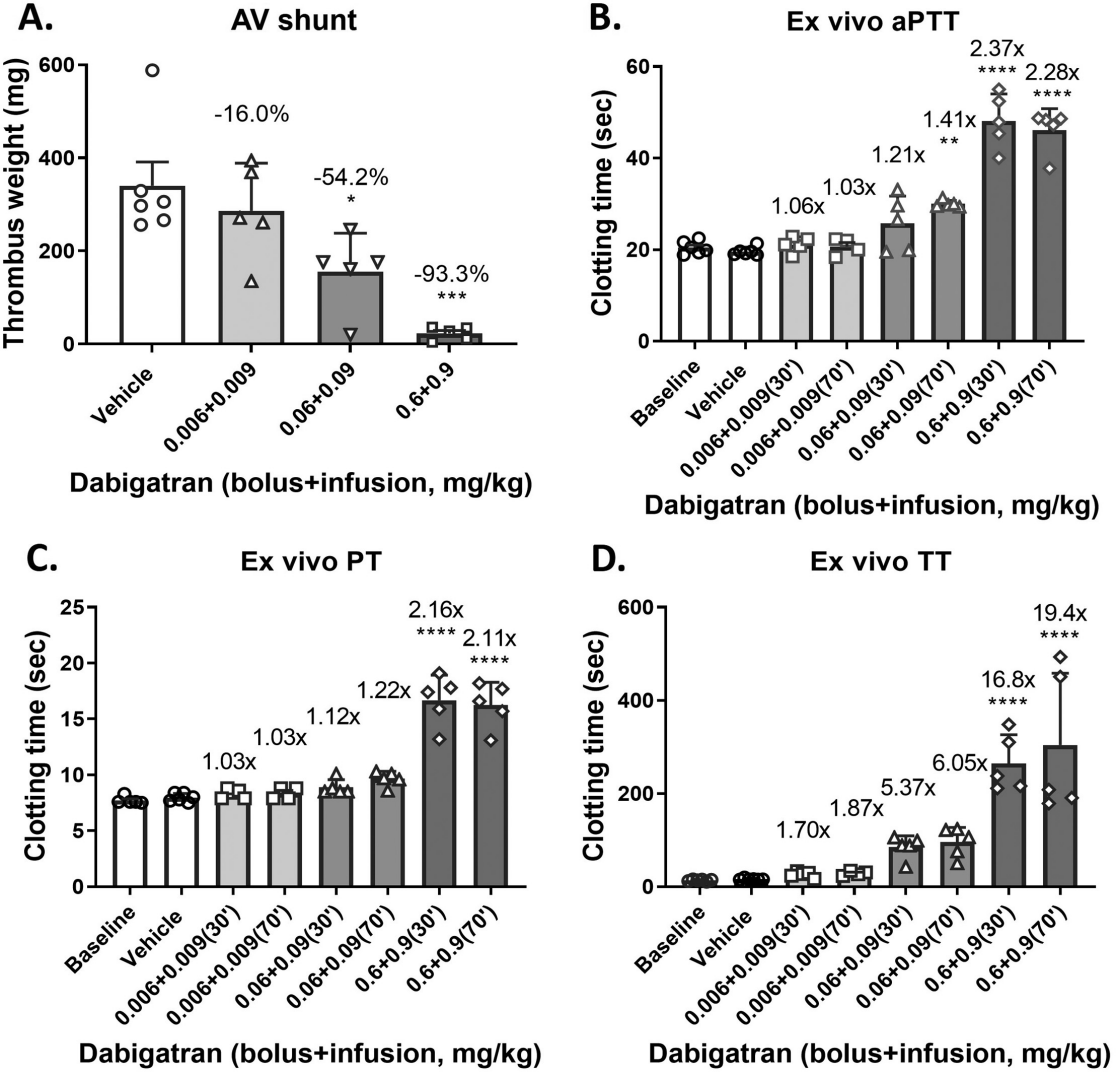
Dose-dependent effects of dabigatran on thrombus formation in the rabbit arteriovenous (AV) shunt model of thrombosis and ex vivo clotting. ( **A** ) Dabigatran dose dependently reduced thrombus weight in the rabbit AV shunt model of thrombosis. Plasma samples were taken immediately prior to (30 minutes after infusion) and after (at 70 minutes) the AV shunt thrombosis and subjected to the ex vivo analysis of ( **B** ) activated partial thromboplastin time (aPTT), ( **C** ) prothrombin time (PT), and ( **D** ) thrombin time (TT). Each symbol in ( **A** ) represents 1 animal. Fold changes are indicated against vehicle in ( **B** ) and ( **C** ). * *p* < 0.05. ** *p* < 0.01. *** *p* < 0.001. **** *p* < 0.0001. Analysis of variance (ANOVA) followed by Tukey's test.

Dabigatran also dose dependently reduced thrombus weight in the rabbit AV shunt model of thrombosis, with 16.0 ± 13.5, 54.2 ± 10.9 ( *p* <0.05; *n* = 5), and 93.3 ± 1.9% ( *p* < 0.001; *n* = 5) inhibition versus vehicle, respectively, at doses of 0.006 + 0.009, 0.06 + 0.09, and 0.6 + 0.9 mg/kg of bolus plus infusion as summarized in Fig. 3A . Ex vivo clotting assays showed significant increases for dabigatran in aPTT (2.37-fold; *p* < 0.0001), PT (2.16-fold; *p* < 0.0001), and TT (16.8-fold; *p* < 0.0001) at the 0.6 + 0.9 mg/kg dose upon AV shunt start ( Fig. 3B–D ).

## Discussion

The rabbit AV shunt model of thrombosis is one of the most widely used animal models to characterize the antithrombotic efficacy of anticoagulants, including all of the currently approved oral anticoagulants. This model employs an extracorporeal perfusion system via contact activation for thrombus formation under different shear rate of blood flow. 30 The thrombus produced by this model is similar to venous thrombus composed of mainly red blood cells and fibrin with some platelets and leukocytes, with the characteristics of human pathology. 31 In the present study, we report that milvexian dose dependently inhibited venous thrombosis. Consistent with its mode of action, milvexian also caused a dose-dependent prolongation of ex vivo aPTT, but not PT or TT, demonstrating selectivity for FXIa inhibition in the intrinsic pathway of coagulation. The plasma drug levels of milvexian were 0.515 ± 0.063, 1.695 ± 0.059, and 5.467 ± 0.533 μM at 0.25 + 0.17, 1.0 + 0.67, and 4.0 ± 2.68 mg/kg bolus + mg/kg/h infusion, respectively.

This study was done in parallel with two reference therapeutic agents, apixaban and dabigatran, as a part of model calibration. Dose-dependent inhibition in thrombus weight was demonstrated for both apixaban and dabigatran. The antithrombotic efficacy of apixaban was previously reported in various rabbit models of thrombosis, including AV shunt thrombosis, DVT, and electrically induced carotid artery thrombosis. 25 29 The effect of apixaban observed in the AV shunt thrombosis model was in agreement with previous reports in rabbits 25 29 and in line with the clinical studies of apixaban for the prevention/treatment of venous thromboembolism. 32 Likewise, the effective dose range in the current study for dabigatran in the rabbit AV shunt model was also in agreement with previous reports for dabigatran in a rabbit model of venous thrombosis. 33

With respect to the coagulation parameters studied, apixaban increased both aPTT and PT, demonstrating its impact on the common pathway of coagulation. Dabigatran also increased aPTT, PT, and, to a larger extent, TT, demonstrating its role in targeting the common pathway and the final step of coagulation. Thus, apixaban and dabigatran are expected to inhibit coagulation initiated by either the intrinsic or extrinsic pathway of coagulation. In contrast, milvexian selectively inhibits coagulation initiated by the intrinsic pathway of coagulation and is not expected to have an appreciable impact on coagulation initiated by the extrinsic pathway of coagulation (deemed critical for hemostasis), as evidenced by a lack of effect on PT. Although bleeding was not assessed in the current study, we have previously reported that milvexian did not increase cuticle bleeding time in rabbits either alone or when added on top of aspirin. 24 In contrast, apixaban and dabigatran have been demonstrated to increase bleeding in rabbit models. 25 29

The AV shunt thrombosis model used in the present study is designed to replicate the key pathophysiologic features of fibrin-rich venous thrombosis. Similar preclinical validation was also recently demonstrated for another FXIa inhibitor, asundexian, in a rabbit AV shunt thrombosis model. 34 More recently, we have reported on the efficacy of milvexian for the prevention of venous thromboembolism in patients undergoing total knee arthroplasty without any statistically significant increases in bleeding compared with enoxaparin. 22 23 In addition to venous thrombosis, the in vivo efficacy of milvexian was demonstrated in a rabbit ECAT model, 24 further supporting clinical evaluation in settings of arterial thrombosis.

It should be pointed out that while no hemostasis studies were conducted for milvexian in the current report, the advantage of FXI/FXIa blockage over direct-acting oral anticoagulants (DOACs) on hemostasis has been provided through various lines of both preclinical and clinical evidence. 3 13 14 15 16 17 35 36 DOACs are effective as anticoagulant therapies, but have been associated with bleeding complications such as intracranial and gastrointestinal bleeding. 35 36 A previous study in a rabbit venous thrombosis model (cuticle bleeding model) revealed that apixaban and dabigatran prolonged bleeding time by 1.13 ± 0.02- and 4.4 ± 0.4-fold, respectively, at doses associated with an ∼80% reduction in thrombus formation. 29 In contrast to DOACs, either FXIa inhibitors or FXI deficiency provided protection from thrombosis with no increase in bleeding times in various species. 3 13 14 15 16 17 Human hemophilia C patients who are severely deficient in FXI have a mild to moderate risk of provoked bleeding and display a reduced incidence of DVT and/or ischemic stroke. 6 7 As expected, no bleeding liability was found for milvexian in the rabbit cuticle bleeding model (milvexian alone and/or in combination with aspirin). 24 Likewise, milvexian demonstrated robust efficacy in a phase 2 clinical study for the prevention of venous thromboembolism in patients undergoing total knee arthroplasty with no statistically significant increase in bleeding. 23

The results of the current study demonstrated that milvexian is an effective anticoagulant for the prevention of venous thrombosis in the rabbit model, which supports the utility of milvexian in venous thrombosis.

## Notes

**What is known about this topic?**

- FXIa is an emerging target for the prevention and treatment of thrombosis.
- Inhibition of FXIa is a promising mechanism to prevent thrombosis while preserving hemostasis.

**What does this paper add?**

- This study demonstrates the antithrombotic effect of milvexian in a rabbit model of venous thrombosis.
